# ‘A recipe for cultural disaster!’– a case study of Woolworths Group’s proposal to build an alcohol megastore in Darwin, Northern Territory

**DOI:** 10.1186/s12992-023-00938-5

**Published:** 2023-06-10

**Authors:** Alessandro Connor Crocetti, Beau Cubillo (Larrakia), Troy Walker (Yorta Yorta), Fiona Mitchell (Mununjali), Yin Paradies (Wakaya), Kathryn Backholer, Jennifer Browne

**Affiliations:** 1 Global Centre for Preventive Health and Nutrition (GLOBE), Institute for Health Transformation, Faculty of Health, Deakin University, Geelong, VIC Australia; 2 Deakin Rural Health, Faculty of Health, Deakin University, Warrnambool, VIC Australia; 3 Deakin University, Alfred Deakin Institute for Citizenship and Globalisation, Burwood, VIC Australia

**Keywords:** Indigenous health, Alcohol industry, Commercial determinants of health, Health equity

## Abstract

**Background:**

The health and wellbeing impacts of commercial activity on Indigenous populations is an emerging field of research. The alcohol industry is a key driver of health and social harms within Australia. In 2016 Woolworths, the largest food and beverage retailer in Australia, proposed to build a Dan Murphy’s alcohol megastore in Darwin, near three ‘dry’ Aboriginal communities. This study examines the tactics used by Woolworths to advance the Dan Murphy’s proposal and understand how civil society action can overcome powerful commercial interests to protect Aboriginal and Torres Strait Islander health and wellbeing.

**Methods:**

Data from 11 interviews with Aboriginal and non-Aboriginal informants were combined with data extracted from media articles and government, non-government and industry documents. Thematic analysis was informed by an adapted corporate health impact assessment framework.

**Results:**

Woolworths employed several strategies including lobbying, political pressure, litigation, and divisive public rhetoric, while ignoring the evidence suggesting the store would increase alcohol-related harm. The advocacy campaign against the proposal highlighted the importance of Aboriginal and non-Aboriginal groups working together to counter commercial interests and the need to champion Aboriginal leadership. Advocacy strategies included elevating the voices of community Elders in the media and corporate activism via Woolworths’ investors.

**Conclusions:**

The strategies used by the coalition of Aboriginal and non-Aboriginal groups may be useful in future advocacy campaigns to safeguard Aboriginal and Torres Strait Islander health and wellbeing from commercial interests.

## Background

Alcohol consumption is a leading contributor to death and disease worldwide, and Australians are among the heaviest drinkers in world [1, 2]. In 2021, Australia recorded 1,559 alcohol-related deaths [3], with 3 out of 5 drug-related hospitalisations attributed to alcohol [4]. At a population level, alcohol consumption is linked to over 30 diseases and injuries, including road traffic accidents, suicide and self-harm, various cancers, cardiovascular, liver and pancreatic diseases. Additionally, it is a major factor in reported incidents of domestic violence and child abuse [5–8]. Of the six states and two territories, the Northern Territory (NT) has the smallest population of all the Australian jurisdictions, with an estimated 250,635 people [9]. Yet the NT has the highest per capita alcohol consumption, ranking second highest in the world [10], and the highest rate of alcohol-related harm in Australia [11].

Aboriginal and Torres Strait Islander peoples, the First Peoples of Australia, have maintained ongoing connections to their lands and knowledge systems for over 65,000 years [12]. These social, cultural and ecological connections are recognised as the oldest continuous civilisation in the world [12]. However, the ongoing impact of settler colonialism on Aboriginal and Torres Strait Islander peoples is contributing to health inequities through dispossession of land and resources, disempowerment, assimilation and political oppression [13, 14]. Proportionally, the NT has the highest Aboriginal and Torres Strait Islander population of all Australian jurisdictions, with 30.8% of Northern Territorians identifying as Aboriginal or Torres Strait Islander, compared to 3.8% of the national population [15]. Aboriginal and Torres Strait Islander people experience a disproportionate burden of alcohol-related harm, despite being more likely than non-Indigenous Australians to abstain from alcohol [16]. Mortality attributable to alcohol is five times higher compared to non-Indigenous Australians [17] and up to ten times higher for Aboriginal people^1^ in the NT compared to the total Australian population [18]. This disparity is one of the contemporary effects of colonisation [19].

Products that are harmful to health (such as alcohol), the companies who sell them, and the strategies they employ to maximise sales and profits are conceptualised as Commercial Determinants of Health (CDoH). A Commercial Determinants of Health (CDoH) lens interrogates the “systems, practices and pathways through which commercial actors drive health and equity” (p.2, [20]). For example, conflict between the alcohol industry and public health objectives was evident in the prolonged battle with the Australian government to delay implementation of mandatory pregnancy warning labels on alcohol products [21]. The consistent playing down of health risks and questioning the credibility of research are common tactics used by the alcohol industry globally [21–23].

Commercial actors employ a “playbook” of strategies and tactics to further their interests, oppose regulation and undermine population health [24]. In the political environment, this may involve, among other corporate political activities, regulatory capture by building powerful relationships with policy makers, providing political donations [25], and lobbying governments [26]. An example of this is the ‘the revolving door’ mechanism whereby past industry representatives secure positions within government agencies responsible for regulation, while previous government employees secure positions within the private sector they were previously regulating [27]. Such corporate political strategies have been documented both in Australia [25, 27] and internationally, including in low and middle-income countries (LMICs) [28–34].

The alcohol industry consists of numerous commercial entities across the production, supply, distribution and retail sectors as well as industry-funded non-government and public relations organisations who appear to promote health messages, such as ‘DrinkWise’ [35], while covertly promoting policies most favourable to their commercial interests [36, 37]. Marketing is another common strategy used by the alcohol industry, who advertise across multiple media platforms and frequently sponsor popular sports and cultural events to normalise and glamorise drinking [38, 39]. There is also evidence of the alcohol industry targeting women, young people and ethnic minorities by directing their marketing campaigns at these population sub-groups [40–43].

Woolworths Group (hereafter referred to as Woolworths) is the largest food and beverage retailer in Australia [44] and together with Aldi and Coles is part of what is known as the supermarket oligopoly [45]. In 2022 these three supermarkets collectively held 75% of the Australian market share [46]. In 2016, Woolworths applied for a license to establish a Dan Murphy’s outlet in Darwin, the capital of the NT [11]. The proposed Dan Murphy’s store was going to have 48 times the amount of alcohol volume of the alcohol outlet it was replacing and was to be within walking distance of three ‘dry’ Aboriginal communities – Kulaluk, Minmarama Park and Bagot, where numerous Aboriginal People groups reside [47]. These are communities that elected to ban the selling or consumption of alcohol [48]. The proposed Dan Murphy’s store led to a 5-year political and legal battle and an advocacy campaign, which culminated in Woolworths pulling out of the development.

The health and wellbeing impacts of commercial activities on Indigenous populations is an emerging field of research, and evidence shows that the alcohol industry negatively impacts Aboriginal health within Australia [16, 18, 49]. There has been extensive research conducted on the impacts of alcohol for Aboriginal communities [16, 18]; however, there have been no specific analyses to understand how the structures and practices of the alcohol industry contribute to health inequity for Aboriginal people. The case of the Woolworths proposal to build a Dan Murphy’s alcohol megastore in the NT is a prime example of the commercial determinants of Indigenous health (CDoIH). It involves a powerful commercial entity implementing activities designed to increase profits that are likely to have a negative impact on Aboriginal health and wellbeing through increased alcohol-related harm [11]. The fact that Woolworths’ plan was abandoned following a long advocacy campaign led by local Aboriginal and non-Aboriginal organisations, provides an important opportunity to understand how community advocacy can overcome powerful commercial interests when it comes to protecting Aboriginal health. In this study, we aimed to use case-study methods to: i) assess the potential health and wellbeing impacts of the proposal by Woolworths Group to build a Dan Murphy’s megastore near three Aboriginal communities in Darwin (NT); ii) examine the tactics used by Woolworths to advance the Dan Murphy’s proposal; and iii) examine how civil society action can overcome powerful commercial interests when it comes to protecting Aboriginal health.

## Methods

### Researcher positionality

This study was led by non-Indigenous Australian doctoral student (AC) who worked alongside Aboriginal (BC, YP) and non-Aboriginal scholars (JB, KB) to co-design a research approach that valued Aboriginal lived realities and resisted colonial perspectives. AC worked closely with an Aboriginal (Larrakia) researcher (BC) to ensure an appropriate data collection and analysis approach was implemented, and to provide Aboriginal visibility within the research process to reduce power imbalances with participants and align coherently with Indigenous peoples’ principles of ethical research [50]. The study was supervised by a senior Aboriginal (Wakaya) researcher (YP) and non-Indigenous researchers with experience in Aboriginal health (JB) and CDoH research (KB), and this article was developed in collaboration with Indigenous co-authors (TW, FM).

### Study design

A qualitative case study approach was employed to examine the proposed Dan Murphy’s store in Darwin and its potential health and wellbeing impacts for local Aboriginal communities. A case study investigates a phenomenon in its real-world context and is particularly useful where the boundaries between the two (the phenomenon and the real-world) may not be clearly elucidated [51]. In this project, the set of institutions, actors, ideas, and events surrounding the Dan Murphy’s proposal, and its ultimate abandonment provide a rich case study of the CDoIH in Australia.

#### Theoretical framework

**Fig. 1:**
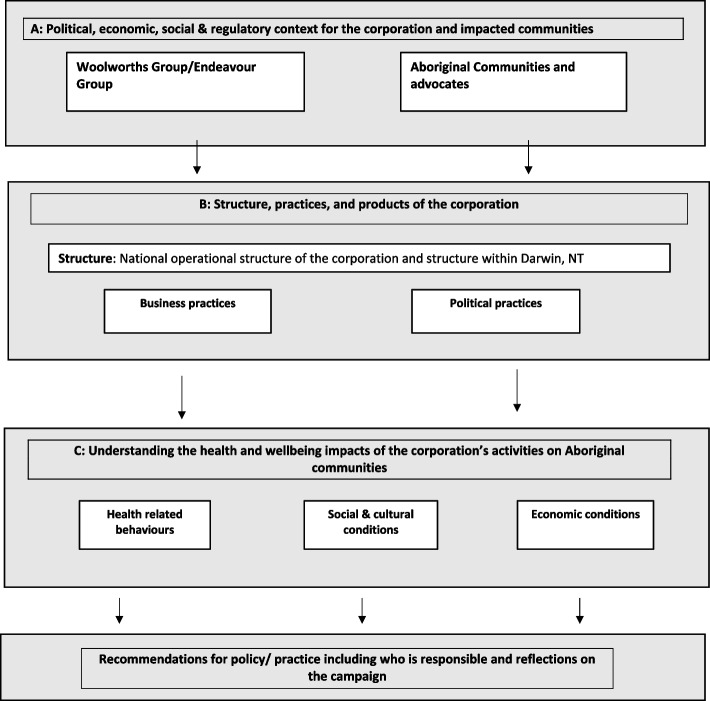
Corporate health impact assessment framework adapted from Baum et al. (2016) [59]

Data collection and analysis were informed by theories and frameworks of the CDoH [24, 52–58]. We additionally adapted a corporate health and social impact assessment (HSIA) framework, developed by Baum and colleagues, to incorporate literature on the cultural aspects of Aboriginal health and wellbeing [59, 60]. We also tailored the framework to assess the impacts of a corporation solely at the national and local level. The HSIA framework was specifically developed for assessing health impacts of transnational corporations and has been applied in several Australian case studies [52, 61, 62]. It examines the structures and practices of commercial actors and has a specific focus on the health equity impacts [59]. Thus, it provides a useful framework to guide our analysis of the practices of a large corporation, such as Woolworths in this case study. The Corporate HSIA framework consists of three elements (Fig. 1). Firstly, it considers the specific context in which the corporation operates. Secondly, it examines the corporation’s structure, products, and political and business practices that have the potential to impact health and wellbeing. Lastly, it explores health and equity impacts of the corporation, encompassing social, economic, and environmental conditions, as well as health-related behaviours [59]. We added cultural wellbeing to the framework to ensure our analysis was sensitive to the Aboriginal concept of health which is “not just the physical well-being of an individual but refers to the social, emotional, and cultural well-being of the whole Community” [59].

##### Data collection

We collected data from multiple sources, as is recommended in case study research [51].

Three types of documents were collected and combined with data from key informant interviews. Industry documents (*n* = 6) were sourced from the Woolworths website, as well as other publicly available websites which contained submissions from Woolworths/Endeavour Group relevant to this case. Government documents and relevant legislation (Liquor Acts) (*n* = 15) were sourced from NT government websites and the parliamentary Hansard. Industry and government documents were sourced using an iterative approach that was informed by keywords relating to the case, specific dates, information from media articles and informants. In September 2022, we sought access under the Northern Territories Rights to Information Act 2002 to any documented correspondence between ministers or senior public servants and external organisations regarding the proposal to build the Dan Murphy’s store in Darwin. Through this process, we received 20 emails from the Department of the Chief Minister and Cabinet and 15 from the Department of Industry, Tourism, and Trade. Non-government documents (*n* = 17) included policy submissions by academic researchers, Aboriginal health organisations and other advocacy organisations. Media articles covering the Dan Murphy’s proposal published in the in Australian news media were sourced from ProQuest Australia and the New Zealand Newsstream database or manually searched from each relevant publication website using the terms ‘woolworths’ and ‘dan murphy’s’ with date range from 2016-present. After initial screening for relevance, 125 media articles were retrieved for analysis.

##### Key informant interviews

Eleven semi-structured interviews were conducted with stakeholders who had been involved in alcohol policy, research, or advocacy in the NT during the case study period (2016–2021). A combination of purposive and snowball sampling was used to recruit participants. Key informants were selected, in the first instance, based on the names identified from media articles and other documents as well as through recommendations from other interview participants. Potential participants were invited via email, with a follow-up email sent if a response was not received within 2 weeks. Through this process, 26 individuals were invited to participate and 11 agreed to participate.

Seven interviews were conducted by AC and four interviews co-facilitated with a Larrakia researcher (BC), to enhance cultural safety for participants. Interviews were undertaken between July and September 2022 and ranged from 20 to 82 min in length. Participants included representatives from non-government organisations (*n* = 4), Aboriginal Community-Controlled Health organisations (*n* = 2), policy advisors (*n* = 2), and health researchers (*n* = 3). Industry representatives were not interviewed as this was this was not approved by the university ethics committee without organisational consent and there are already multiple statements from Woolworths about this case in the public domain. Two interviews were conducted in person, and the remaining 9 interviews were conducted over Zoom. Participants were asked questions in relation to the series of events that occurred over the course of the proposal and the campaign against it (section A), the perceived strategies used by Woolworths (section B), the key players involved, and the potential health and wellbeing impacts of the proposed Dan Murphy’s development may have had on local Aboriginal communities (Section C). Participants were also asked to reflect on the success on the community-led advocacy campaign and to provide recommendations for future policy and advocacy practice.

### Data management and analysis

Documents were saved into electronic folders, organised chronologically and by the type of data (e.g. industry, government, non-government organisation documents or media articles) as recommended in case study research [51]. Documents were used to create a narrative timeline of the key events and processes surrounding the case study. Audio recordings of interviews were transcribed and uploaded along with media articles, industry, government, and non-government documents into NVivo 20 qualitative analysis software (QSR international). The data were initially coded inductively by one investigator (AC) and codes were grouped together to generate themes [63]. Themes were then organised under each of the categories of HSIA framework. Codes were sense-checked by an Aboriginal investigator (BC) and differences in interpretation were resolved through discussion. Interview findings were corroborated with findings from documentary data. The iterative process of data collection, transcription, analysis and constant comparison across multiple data sources and investigators enabled reflexivity when deriving meaning from the data.

### Ethics

This study was conducted according to the guidelines for ethical conduct in Aboriginal and Torres Strait Islander health research [64] and approved by the Deakin University Human Research Ethics Committee (approval number 2022-105). Written informed consent was obtained from all interview participants.

## Results

In the following sections we provide an overview of the key themes derived from the data, organised under sections A, B and C of the corporate HSIA framework: a) the political, social economic and regulatory context, b) structure, practices and products, and c) health and wellbeing impacts. A timeline of key regulatory events can be found in Table 1. Illustrative quotations from documents and interview participants are provided to support key findings. Participant codes and types are provided in brackets following each quotation.

**Table 1:** Timeline of regulatory and legal events, adapted from FARE (2021) and Gilbert (2021) [65, 66]

**Date**	**Event**
December 2016	NT Liquor Regulations amended capping the maximum retail floor space at 400 square metres [67].
December 2016	Woolworths applies for alcohol license substitution for Dan Murphy’s store
March 2017	Woolworths takes legal action questioning the regulatory powers of the Government at the Federal Court of Australia
March 2017	NT Alcohol Policies and Legislation Review panel announced [68].
August 2017	Woolworths withdraws case from the Federal court
October 2017	Alcohol Harm Reduction Act 2017 introduced based on recommendations of Alcohol Policies and Legislation Review [69]
February 2018	NT Independent Liquor Commission re-established to regulate liquor licencing in the NT [70, 71]
June 2019	Woolworths applies for alcohol substitution licence to swap a small alcohol outlet in Darwin for a large alcohol outlet close to three ‘dry’ Aboriginal communities
September 2019	Liquor Commission denied application on the grounds that the new premises did not yet exist, and this was a requirement for licence substitution within the Act [47].
October 2019	New Liquor Act 2019 [72] introduced. Woolworths appeals Liquor Commission decision at the Northern Territory Civil and Administrative Tribunal (NTCAT)
December 2019	NTCAT denies Woolworths’ appeal
January 2020	Woolworths takes case to Supreme Court of the NT
March 2020	Liquor Amendment Bill 2020 [73] allows Liquor Commission to approve substitution of premises. when the proposed premises are not yet to be constructed
April 2020	Woolworths re-appeals the case at NTCAT
November 2020	Liquor Further Amendment Act 2020 [74] introduced, giving decision-making power on alcohol licenses to the Director of Liquor Licensing instead of Liquor Commission
December 2020	Director of Liquor Licensing grants approval of Woolworths’ application
February 2021	Woolworths board establishes Independent Panel Review (Gilbert Review). 138 stakeholder submissions received [66].
April 2021	Gilbert Review report recommends Woolworths do not proceed, citing inadequate consultation with Aboriginal communities [66]. Woolworths abandons plan
July 2021	Woolworths announces demerger with Endeavour Group [75].

### Part A – political, social, economic, and regulatory context

#### Socio-economic, socio-political, and cultural landscape

Darwin is a regional centre, located on the traditional lands of the Larrakia people. It has a population of 139,000 people, 10.4% of whom identify as Aboriginal [76]. There were approximately 357 residents in the three Aboriginal communities within 1.5 km of the proposed Dan Murphy’s site – Bagot [77], Minmarama Park [78] and Kulaluk [79], with residents coming in and out depending on the season. Residents in these communities experience social and economic disadvantage, with median household income one - third that of Darwin city [80].

The normalisation and strong acceptability of alcohol in Darwin was discussed by several interview participants. The fact that Territorians *“out drink every other part of Australia”* (non-government organisation representative #3) was a key concern for those working in the health sector. Participants who were involved in the campaign against the Dan Murphy’s store explained there was already a vast amount of alcohol in Darwin, with many outlets already in place so that, even without the Dan Murphy’s store, there are many opportunities for Darwin residents to purchase alcohol.

“Darwin is a city saturated with liquor licenses, there is no shortage of alcohol in Darwin […] people in Darwin aren’t missing out on alcohol should that be something they wish to procure” (non-government organisation representative #4)

### Political and regulatory context

The NT has been governed by the Australian Labor Party since 2016. Shortly after their election, the Labor government commissioned the Alcohol Policies and Legislation Review [68]. The alcohol policy landscape in the NT has been through a series of rapid changes over the last decade. One participant described alcohol policy in the NT as a *“political football”* (researcher #1) which can be partially attributed to the changing of governments and the increased “politicisation of alcohol policy” [19]. One alcohol researcher outlined the instability in the alcohol policy landscape as follows:

“If you look at a timeline of alcohol policies that have been in place in Darwin since 2013 it’s just insane how much change there has been, so one government will put something in place and it will be repealed, then they try something different then go back doing the old thing – so there’s this really long history of chop-and-change-chop-and – change policies.” (researcher #1)

The strong consensus from participants was that, over the last 30 years, governments in the NT have been heavily populist, focusing on issues that garner attention in the media. The main newspaper in the NT, *The NT News,* owned by conservative media company News Corp Australia [81, 82], frequently publishes stories about politics, business and personal financial prosperity. The wider public and political discourse in the NT focussed on building a stronger economy and upholding individual freedoms. One participant summarised the popular rhetoric as “*alcohol harm is a problem, but we should be able to enjoy a beer,” (researcher #2)*. The political tension between protecting public health and supporting the local economy is exemplified in this quote from a policy advisor:

“Government probably had the view that initially we’ve got to do something about alcohol, and we’ve got to stick up for local businesses and they could see it becoming such a hot political issue” (policy advisor #1)

After establishing an Independent Liquor commission to oversee alcohol licencing in the NT*,* in November 2020 the government “*moved legislation forward to give the director of licensing the powers to approve the application” (Aboriginal community-controlled health organisation representative #1)* thus by-passing the independent regulatory process and leading to Woolworths’ license being approved (December 2020). A more detailed timeline and overview of the key legal/ regulatory events can be found in Table 1.

### Part B—Woolworths structure and practices

#### Woolworths structure and history

In this case study, some of Woolworths’ activities in relation to the Dan Murphy’s proposal were undertaken by its beverage and hotel business arm, Endeavour Group. Endeavour Group was created in 2020, following a corporate restructure, and oversaw Woolworths’ alcohol business activities until their demerger in July 2021 [75]. Endeavour Group owns Dan Murphy’s, one of Australia’s largest alcohol retailers, making $AUD 9.28 billion in sales in 2020 [83]. There are over 250 Dan Murphy’s stores across Australia, representing 20% of the Australian retail liquor market, but none in the NT [66, 84]. As Woolworths’ owned Endeavour Group for most of the period of this case study, the commercial entity is referred to as Woolworths throughout this article.

Woolworths has a long history, dating back to the 1990s of inadequate community engagement or being in direct conflict with communities when it comes to alcohol developments. Interview participants, evidence from submissions to the NT Liquor Commission [85, 86] and the report of the independent review commissioned by Woolworths [66] provided examples, from across Australia, of communities fighting against Woolworths’ alcohol outlets. Such conflicts with local communities occurred particularly in areas with high levels of alcohol-related harm, including other towns with high Aboriginal populations such as Derby in Western Australia [87], Nhulunbuy and Katherine, in the NT [88–90]. In previous cases, Woolworths was described as being very persistent in the face of opposition, as one participant described “*Woolworths ducked and weaved” (non-government organisation representative #2)* its way around various regulatory hurdles until the licence was approved. Another participant summarised Woolworths conduct as follows:

“Woolworths does not have a good track record in terms of engaging communities and when they do engage communities, they do what they want anyway” (researcher #2)

#### Political practices and strategies

##### Litigation

Woolworths used several different business practices and strategies throughout its campaign to build a Dan Murphy’s store in Darwin. This included legal action, such as taking the NT government to court after it amended the liquor regulations reducing the maximum retail floor space for alcohol outlets to 400 square metres (Liquor Amendment Regulations 2016). Woolworths also appealed the Independent Liquor Commission’s decision to deny its application for a substitution of alcohol license. The appeal first occurred via the NT Civil and Administrative Tribunal (NTCAT) (October 2019), and then taking the case to the Supreme Court of the NT following NTCAT denying the appeal (January 2020). Ultimately, legislation was changed to grant the director of liquor licensing power to approve the proposal (Liquor Further Amendment Act 2020).

The legal battle between Woolworths and the NT government was described by participants as a long and drawn-out process. Advocates noted the vast resources Woolworths had at their disposal, which enabled them to employ the *“last man standing strategy”* (non-government organisation representative #1) by continually appealing government decisions *“when the politics wasn’t right for them”* (non-government organisation representative #2). Participants characterised the multiple appeals and legal threats made by Woolworths when *“they didn’t like what the government did”* (non-government organisation representative #1) as an attempt to wear down the opposition until their application was approved.

“They had all the evidence that the application shouldn’t proceed due to the potential of community harm – everyone thought it was done and dusted, but they persisted with a well-funded legal battle to have the decision overturned.” (researcher #2)

Respondents also mentioned the deceptive public discourse used by Woolworths during the litigation process. One participant described how Woolworths would tell the media that “*the government’s taking too long to make a decision” (non-government organisation representative #1)* when the application had already been rejected by the government. Another participant reported that Woolworths would use delay tactics to limit the number of scientifically rigorous submissions that could be provided by those opposing its application to counter Woolworths’ arguments.

“Woolworths repeatedly left it to the 11th hour to submit anything to the Liquor Commission and or other bodies at the time which gave little windows of time for responses to be prepared.” (researcher #2)

##### Lobbying and political pressure

Many interview participants, speculated that Woolworths were aggressively lobbying the NT government to approve their application. A number of documents reinforced this finding, with the Independent Panel Review recommending that *“Woolworths Group commits to reviewing the way in which it engages with governments on future business plans”* (p.91, [66]). Interviewees reported that Woolworths used a *“crash through approach*” *(Aboriginal community-controlled health organisation representative #2)* and that “*they were clearly putting pressure on the government”* (non-government organisation representative #1) to change the liquor licencing laws. Documents received under Freedom of Information confirmed that Woolworths was in regular contact with the director of liquor licensing, and the offices of the Health Minister and Chief Minister with regard to the Dan Murphy’s proposal and associated policies. Participants noted the change in political rhetoric surrounding the matter before legislation was swiftly passed to allow for Woolworths to receive the license. With government ministers, including the Minister for Small Business explaining to the press that this legislation will further the governments *“red-tape busting agenda”* (The Australian Broadcasting Corporation, 11/11/2020, Paragraph 4). One interview respondent suggested:

“[Woolworths] aided and abetted with the NT government inappropriately…they persuaded or partly persuaded the territory government to amend the legislation to facilitate the license approval” (policy advisor #2)

##### Media strategy

*The NT News* has an estimated readership of 397,000 people per month [91]. Its coverage of the Dan Murphy’s application, and associated political and legal processes, was overtly in favour of Woolworths. The NT News published headlines including “Bitter blow as no Dan Murphy’s for Darwin” (21/09/2019) when the application was initially denied; “It’s time to approve Dan Murphy’s” (13/11/2020) during the director of liquor licensing hearing; and “Here’s cheers to final nod for Dan Murphy’s” (18/12/2020) when the licence was ultimately granted. Interview participants suggested that the *“ongoing media hype”* (policy advisor #1) including news stories reporting that most residents were in favour of the Dan Murphy’s, was potentially a strategy by Woolworths.

“One of the biggest challenges that we face these days is our media is bought lock stock and barrel by the alcohol industry” (researcher #3)

At the national level, media publications including The Guardian were supportive of the community advocacy efforts stating that local Aboriginal health *“organisations do not support putting one of the biggest bottle shops in Australia within walking distance of three ‘dry’ Aboriginal communities”* (13/11/2020).

#### Business practices and strategies

##### Community engagement and consultation

An ongoing dispute between Woolworths and community advocates throughout the campaign was the selective nature of community consultation undertaken by the company. According to interview participants, key Aboriginal health organisations, other health bodies and Aboriginal communities were not involved. For example, one Aboriginal organisation representative expressed that *“it was all about profit-making as opposed to having a social conscience”* (Aboriginal community-controlled health organisation representative #1). Lack of appropriate consultation was also highlighted in the report of the independent review into the process that Woolworths commissioned:

“The failure from the outset to identify, engage with and listen to the broad range of stakeholders concerned with the impact of a Dan Murphy’s development on Aboriginal and Torres Strait Islander peoples of Darwin and the inadequate consultation processes that were employed.” (p.127, [66])

Conversely, Woolworths continually argued that they had, in fact, undertaken proper community consultation and engagement. A spokesperson from Woolworths suggested in a news article that, according to the company’s polling, “*over 80 percent of people in Darwin support a Dan Murphy’s store opening”* (The Geelong Advertiser, 18/12/2020, paragraph 19). Yet, interview participants reported the issue was much broader than one of community engagement. They suggested that Woolworths actively avoided the key health bodies in the NT and *“sought to minimise the knowledge and expertise of the health people”* (policy advisor #1). Critically, this included Aboriginal health organisations, as this advocate explained:

“They didn’t talk to us, they didn’t even acknowledge our presence[...]there was no engagement and that continued all the way through” (Aboriginal community-controlled health organisation representative #2)

Civil society advocates described the tactics that Woolworths was using to get its application approved. They reported that Woolworths *“treated the community with disdain”* (non-government organisation representative #1) in response to opposition from local Aboriginal organisations and communities. Woolworths was also characterised as using divisive rhetoric in order to polarise and distract their opponents. They even went as far to presume to know what Aboriginal people wanted stating at the 2020 Woolworths Annual General Meeting that *“there is no opposition from these Indigenous people” (Woolworths AGM, 12/11/2020)*, and that Woolworths is *“saying this on behalf of the Indigenous people that they had been in negotiation with” (Woolworths AGM, 12/11/2020)*. They also claimed that Aboriginal organisations did not represent the views of local Aboriginal communities, as expressed by this non-government organisation representative:

“It went like this: ‘organisations like (insert name) don’t speak for Aboriginal people[…] and these Aboriginal people over here disagree with you and so they were driving a wedge in the community” (non-government organisation representative #2)

### Profiting from addiction

The alcohol industry relies and capitalises on the heavy drinking minority and promotes the “*glamorization of alcohol*” (policy advisor #2). At the same time Woolworths justified the need for a Dan Murphy’s “to raise the overall standard of service” in Darwin (p.37, [66]). For example, NT Tourism encourages the normalisation of alcohol through social media marketing promoting the world’s longest pub crawl [92] and the signature alcoholic drinks to have in the NT [93]. Despite, the sophisticated marketing, the majority of alcohol is consumed by people who are alcohol dependent, making addiction part of the industry’s business model, as articulated by this interview participant.

“When it comes to alcohol, we know that 10 percent of the people drink 50 percent of the alcohol and 20 percent of the people drink 75 percent of the alcohol so for these alcohol companies to make a profit they have to be addiction industries” (non-government organisation representative #2)

### Part C—health and wellbeing impacts

#### Alcohol-related harm

Almost all key informants reported that, had the Dan Murphy’s store gone ahead, it would have contributed to “*significant harm in the community*” (policy advisor #1). Evidence presented in the various submissions by health experts to government hearings and reviews clearly demonstrated that an increase in the density of alcohol supplied would lead to increases in alcohol-related violence, including domestic and family violence [94–96]. It was also argued that the NT also has the highest rates of road accidents and mortality involving blood alcohol concentrations above the legal limit [68]. Evidence retrieved from submissions also stressed that Aboriginal people are disproportionately negatively impacted by alcohol through increases in alcohol-related conditions contributing to death and morbidity including diseases of the liver, cerebrovascular diseases, alcohol-poisoning, intentional self-harm and transport accidents [97, 98]. Although much of the debate around the Dan Murphy’s proposal was framed as an ‘Aboriginal issue’, those working in alcohol policy cautioned that this is forgetting the wider societal impact of alcohol consumption in the NT. As one interview participant explained:

“Dan Murphy’s profits will come from the rest of the Territorians who still drink massively, at massively higher rates than the rest of Australia” (researcher #3).

Alcohol researchers emphasised alcohol supply as “*a major factor in terms of the amount that people consume*” (researcher #1). Participants argued that, by increasing the alcohol supply, the Dan Murphy’s store would increase the negative health and social impacts of alcohol. Nevertheless, participants also reported that, throughout their campaign Woolworths continually *“manipulated the conversation and ignored the harms”* (non-government organisation representative #1). Concerns about alcohol-related harms were reiterated by public health experts, Aboriginal health representatives and policy advisors. In addition to the health effects of alcohol consumption, participants were also worried about the impact increased alcohol supply would have on community safety, particularly “*increased alcohol-related crime and alcohol-related violence”* (Aboriginal community-controlled health organisation representative #2). As one interview participant suggested:

“It would not have done anything to support communities and provide safety and security to people living around, not just people living in Aboriginal communities but the broader Darwin community as well” (policy advisor #1)

Another layer of alcohol-related harms emphasised by Aboriginal participants was the impact the Dan Murphy’s store could have on cultural wellbeing. Participants explained how increasing alcohol access to local Aboriginal communities who already had high level of alcohol-related harm would interrupt family connections and the passage of cultural knowledge between generations if *“children are left wandering around the community because their parents are off drinking”,* (Aboriginal community controlled health organisation representative #2). Concerns about alcohol-related deaths and potential loss of culture prompted several community Elders to speak out against Woolworths’ proposal stating that *“there is so much domestic violence and children are not getting looked after” (Aboriginal community Elder)*. As another interview participant explained:

“Like what the old people are saying – it would’ve been a recipe for cultural disaster! (Aboriginal community-controlled health organisation representative #1)

#### Economic impacts

One of the main arguments prosecuted by Woolworths was the potential economic benefit that the store could provide for Darwin. This included *“creating 40 new permanent jobs”* (The Australian Broadcasting Corporation, 26/02/2019, paragraph 15) and that it would *“inject $30 million into the community”* (The Australian Financial Review, 12/12/2019). The benefits to the NT economy were also echoed by the NT government with the Minister for Small Business declaring that *“businesses need certainty”* (NT Government, 11/11/2020) when endorsing the legislative amendment that gave the director of liquor licensing decision-making power to approve Woolworths’ application. However, evidence submitted to the independent liquor commission hearing highlighted that the social costs of alcohol would be much higher if the store was built. Alcohol-related harms were estimated to cost the NT community $1.38 billion in 2015. This figure includes harms associated with alcohol attributable premature mortality, road crash costs, alcohol attributable crime, hospital morbidity, health care costs, and child protection costs [99]. As one interview participant highlighted, any economic benefits of a new alcohol outlet would come with a high degree of social cost.

“The Dan Murphy’s line was – everybody deserves choice, we deserve to come here, we’re gonna create jobs, jobs, jobs…we kept saying, at what social cost?” (non-government organisation representative #3)

### Avenues of advocacy

The advocacy coalition against the Woolworths/Dan Murphy’s proposal employed a *“multipronged attack campaign*” (Aboriginal community-controlled health organisation representative #1). They used a comprehensive strategy by providing submissions to the different government hearings, engaging in corporate campaigns, and executed a coordinated media strategy. See Table 2 for a detailed timeline of key advocacy events. The Foundation for Alcohol Research and Education (FARE), a key advocacy organisation, directly confronted the Woolworths chairman and CEO at their 2019, 2020 and 2021 Annual General Meetings asking them to abandon the plans to build the Dan Murphy’s due to the harm it would cause and, at the 2021 meeting, asked whether Woolworths Group planned to address the systemic corporate governance issues outlined in the independent panel review established by Woolworths. FARE also wrote to the Woolworths and Endeavour Group boards emphasising *“the harm the proposed development will cause to local Aboriginal communities*” as well as *“the reputational risk to Woolworths if it continues to proceed*” (p.1, [100]).

**Table 2:** Summary of key dates for advocacy adapted from the Foundation of Alcohol Research and Education (FARE, 2021) [65]

**Date**	**Advocacy strategy**
June 2019	Health organisations, researchers and community advocates provide submissions and evidence to Independent Liquor Commission hearing
August-Oct 2019	Health organisations provide submissions to NTCAT. FARE file an application to NTCAT emphasising application for substitution of license require an existing premises
December 2019	FARE confronts Woolworths chairman at the AGM about the increased risk of alcohol-related harm if the Dan Murphy’s store was built
March 2020	FARE writes to the board of Woolworths Group and to major corporate shareholders
May 2020	Elder, Aunty Helen Fejo-Frith’s plea to stop the Dan Murphy’s released in several media outlets along with videos denouncing the actions of Woolworths by Aboriginal community members
November 2020	Change.org petition receives over 130,000 signatures [101].
November 2020	Health organisations provide submissions to director of liquor licensing hearing
November 2020	FARE confronts Woolworths Group at the 2020 AGM about Aboriginal health organisations and Communities denouncing of the proposed Dan Murphy’s store
December 2020	Forty-five Aboriginal and non-Aboriginal organisations publish open letter to chairman of Woolworths in the Australian Financial Review newspaper
February 2021	Push for NAIDOC and Reconciliation Australia to reconsider support for Woolworths
February 2021	Health organisations, researchers and community advocates provide submissions and evidence to the Independent Panel Review (Gilbert Review)
March 2021	Danila Dilba Aboriginal Health Service challenges Woolworths’ licence approval in the NT Supreme Court
April 2021	Aboriginal and non-Aboriginal community leaders emphasise the increased alcohol-related harm that would come from the Dan Murphy’s in Australian news media outlets including Special Broadcasting Service, NITV and Australian Broadcasting Corporation

A crucial aspect of the advocacy campaign was amplifying the voices of Aboriginal Elders and community members who were likely to be affected by the new Dan Murphy’s store. This media advocacy was important as it elevated what was a territory issue into a national issue. This included hearing the personal stories of alcohol-related harm from local Aboriginal community members and putting a human face on the advocacy effort. In addition, a Change.org petition initiated by an Aboriginal woman gained 130,000 signatures against the proposal, and national Aboriginal organisations, including Reconciliation Australia, supported the coalition of local organisation advocating against Woolworths. This support was highlighted in an open letter to the chair of Woolworths published in the Australian Financial Review December 2020 signed by local and national organisations. As one participant described, utilising the voices of community Elders was particularly powerful in generating national attention towards the issue.

“It’s hard to ignore when you see a video of women particularly talking about family members that have died, suicide and violence that has occurred” (non-government organisation representative #3)

Aboriginal leadership was another incredibly important aspect of the advocacy campaign. Participants particularly emphasised the importance of Aboriginal and non-Aboriginal organisations working together and *“demonstrating to the community that alcohol impacts everyone”* (non-government organisation representative #3). Aboriginal leadership was also exemplified by using wider connections through approaching national organisations such Reconciliation Australia and scrutinising Woolworths’ Reconciliation Action Plan as “*they were not acting in accordance with reconciliation principles”* (Aboriginal community-controlled health organisation representative #2).

A key pivot point in the campaign was when the advocacy strategy switched *“from a legal advocacy to a corporate campaigning space”* (non-government organisation representative #2). One advocate explained it is easy to *“run out the budget on a legal strategy” (*non-government organisation representative #4) especially when faced with *“a large company with unlimited resources”* (non-government organisation representative #3). The corporate advocacy involved advocates buying strategic shares in Woolworths stocks so they could take shareholder action, attend Woolworths’ Annual General Meetings, and engage directly with the board of directors. This process of putting Woolworths’ directors on the spot around their corporate social responsibility duties and the impact that building the Dan Murphy’s would have on the company’s reputation and, importantly, alcohol-related harm in the NT, is described by one non-government organisation representative:

“It was no longer a faceless corporation facing the community it was [Woolworths chair] facing the community and it was each individual board director who was facing the community (non-government organisation representative #2)

Another component of the corporate campaigning was to ‘follow the money’ in order to put pressure on Woolworths via its investors. This involved contacting major shareholders of Woolworths, including investment banks and superannuation funds, letting them know that *“what’s happening is terrible corporate behaviour and this shouldn’t happen”* (non-government representative #2). This strategy proved to be very effective in shifting shareholder opinions against Woolworths, particularly the female investors that *“really understood the impact that this was gonna have – particularly on families and children”* (Aboriginal community-controlled health organisation representative #1).

### A unified voice

When asked about the success of the advocacy campaign, interview participants stressed the importance of *“singing to the same song sheet”* (researcher #2). Additionally, having a coalition of Aboriginal and non-Aboriginal health and civil society organisations advocating against the Dan Murphy’s store through a unified, structured, coordinated, and collaborative effort was viewed as a key strength. The importance building an advocacy coalition with arguments underpinned by evidence and data was expressed by this Aboriginal health advocate:

“Form alliances with other concerned groups, individuals and other organisation that express concern and make sure that your concerns are addressed preferably with evidence” (Aboriginal community-controlled health organisation representative #1)

### Avoiding reputational damage

After a 5-year campaign, Woolworths ultimately pulled out of the Dan Murphy’s development after an independent review, established by Woolworths, recommended to not proceed. The Independent review, which was overseen by a well-known lawyer, cited *“strong concerns about the proposal […] most importantly but not only, Aboriginal and Torres Strait Islander people, and the negative impact on Woolworths Group”* (p.5, [66]). However, the review panel also state that they were *“unable to make an objective assessment of whether a Dan Murphy’s development would increase the overall level of sales, or the volume of liquor consumed” (p.133, *[66]*),* two points that were argued by health experts and community advocates. One of the key recommendations from the review surrounded corporate reputation and the need for corporations to understand their social duties and responsibilities to consumers and other stakeholders, including Aboriginal peak health bodies and other health organisations, therefore

“before making any business decision that may particularly impact Aboriginal and Torres Strait islander Peoples, Woolworths Group engages with relevant groups at a very early point, and after taking advice from leading community members as to how that engagement should be framed and developed.” (p.132, [66])

Participants reflected on the campaign stressing the need for companies to be more socially responsible, emphasising the need for advocates to shed light on the harmful practices of corporations. The fact that Woolworths abandoned their plan, despite a prolonged legal battle to have their application approved was seen as a victory for grass-roots advocacy in the face of corporate power, as this participant explained:

“Communities have more power than they’ve ever had before, the fact that the change.org petition got one-hundred and fifty thousand supporters that’s massive and so even a company like Woolworths can’t ignore that.” (non-government organisation representative #2)

## Discussion

To the best of our knowledge, this case study of a proposed alcohol megastore in Darwin is, the first analysis of the impacts of corporate activities on Aboriginal health and wellbeing in Australia using a CDoH framework. Analysis of key documents and interviews with Aboriginal and non-Aboriginal stakeholders who had been directly involved in alcohol policy and advocacy in the NT revealed contextual, commercial, and public health insights related to the Woolworths’ proposal and its ultimate abandonment. Our findings suggest that a Dan Murphy’s store in Darwin would likely increase alcohol-related harm, not only in Aboriginal communities but also for the wider Darwin population. Despite prolonged lobbying, litigation and political pressure from Woolworths, we also demonstrate how the campaign against the proposal was ultimately successful, which highlights the importance of Aboriginal-led coalitions and elevating Elders’ voices, as well as the role of shareholder activism in public health advocacy. These findings may be useful for other Aboriginal organisations and communities opposing powerful commercial interests.

Woolworths’ proposal was enabled by a neoliberal socio-political environment which favours economic growth and individual liberty and in which alcohol consumption is normalised. As owners (at the time) of the largest alcohol retailers in Australia, Woolworths acted like any commercial company would in their endeavour to capture more market share and maximise profits for shareholders [102, 103]. What was unique in this case was that regulatory environment for alcohol licencing in the NT changed significantly during the case study period. After the application for the Dan Murphy’s store was rejected by the liquor commission in 2019 due to a technicality surrounding the substitution of a license to an outlet that had not been constructed, legislation was amended to allow the approval of licenses that are yet to be constructed. Alcohol legislation was then further amended by allowing for an expedited process, by-passing the independent liquor commission, and giving decision-making power to the chief regulator, the director of liquor licensing. The rapid change in legislation during the campaign reinforce previous findings surrounding the NT government and the increased politicisation of alcohol [19]. The NT government’s decision to approve the liquor license and the increased politicisation of the issue by local newspaper, The NT News, exemplifies the prioritisation of short term political and economic gain at the expense of public health.

The coverage of the case by The NT News was clearly in favour of the Dan Murphy’s development and the stories were framed from the perspective of Woolworths rather than the Aboriginal communities that would likely be affected. Conversely, coverage by The Guardian framed Woolworths’ actions in a negative light and gave voice to the Aboriginal organisations campaigning against the proposal. This finding is reinforced by our recent media analysis, which found that News Corp owned publications were more likely to privilege voices of commercial actors than Aboriginal organisations and communities, while the Guardian was more inclusive of Indigenous voices [104].

The utilization of political and discursive tactics to evade regulation, as observed in this case study, has been documented in previous studies of the alcohol industry [105, 106]. The commercial determinants of health literature suggests that unhealthy commodity industries contribute to global health inequity, both between and within countries by externalising their harms to disadvantaged population groups and low and middle-income countries to maximise profits for the wealthy minority [20, 107]. This case study provides a unique example from within a high-income country where the alcohol industry’s harms would be externalised to the Aboriginal community.

The public health impacts of alcohol are widespread and affect all aspects of society from the individual, to family and to the wider community [108]. As with other industries associated with addiction, including tobacco, pharmaceutical and gambling, the alcohol industry relies and capitalises on “the heavy drinking minority” to make a profit (p.662, [109]). As reported by interview participants, the heaviest drinking ten percent of the population drink more than half of all the alcohol consumed in Australia, and the heaviest drinking 20 percent consume three-quarters of Australia’s alcohol [110, 111]. Heavy drinkers are more likely to live in regional and remote Australia and purchase cheap alcohol [74]. Thus, it is likely that an alcohol megastore in Darwin would likely accelerate the supply of cheap alcohol to heavy drinkers. This risk was underplayed in the public discourse by Woolworths and the NT government throughout legal proceedings, reinforcing the normalization of alcohol in Darwin. Ultimately, the purpose of using such strategies for commercial gain is to deflect attention away from the industry’s involvement in causing harm [112].

A novel action taken by Woolworths was the independent review into the Dan Murphy’s development following the approval of the license. This independent review focused on the adequacy of stakeholder consultation, the extent to which stakeholder concerns were addressed and best practice in the supply and sale of alcohol in the context of Aboriginal and Torres Strait Islander Peoples [66]. While the review may have been a strategy to minimise reputational damage (a risk that was highlighted in the review panel’s report) [66], the fact that the review was requested after the licence had been approved nonetheless highlights the impact of the community advocacy. Corporate reputation is a “dynamic construct” heavily influenced by external forces such as customer perceptions which are inherently constantly in flux [113, 114]. Corporate reputation is co-created by both an organisation and their stakeholders, including the customers and shareholders [113]. The review findings highlighted a critical issue: the lack of adequate consultation by Woolworths with Aboriginal organisations and communities in Darwin, despite the probablity that they would be disproportionately affected by the alcohol outlet [66].

### Implications for business

Companies should adopt a more holistic perspective of who their stakeholders are when making business decisions [113, 115]. In contrast to a ‘maximising shareholder value’ ideology, stakeholder capitalism is a system that focuses on creating long-term trust with all stakeholders over maximising profits [116]. As a company that has committed to a Reconciliation Action Plan (RAP), Woolworths has a responsibility to engage respectfully with Aboriginal stakeholders. RAPs are voluntary processes that outline a company’s desire to foster relationships with Aboriginal and Torres Strait Islander Peoples through cultural awareness, Indigenous employment and procurement activities [66, 117]. In 2021, over 2200 Australian organisations, including Woolworths, had RAPs [118]. Nevertheless, the monitoring and evaluation RAP commitments is insufficient and there is little evidence that RAPs prevent corporate actors from causing harm to Aboriginal communities [119, 120]. In 2022, following the demerger with Woolworths, Endeavour Group, lunched its first emphasising its “aim of building trust, respect and relationships with Indigenous communities, organisations and other stakeholders” (p.3, [121]). Whether this is simply a reputation management strategy remains to be seen; however, we suggest that the community backlash associated with the Dan Murphy’s proposal may have influenced Endeavour’s decision to develop a RAP.

By not adequately consulting with or addressing the concerns of Aboriginal Elders, organisations and residents, Woolworths did not support Aboriginal cultural protocols or self-determination. The right to self-determination is asserted in the United Declaration on the rights of Indigenous peoples, to which Australia is a signatory. It is also a cultural determinant of health required to overcome “historical and ongoing trauma of colonization” and includes both “personal and community empowerment” (p. 2, [122]). The actions of Woolworths and the NT government undermine community-led responses in the NT to reduce alcohol-related harm including night patrols [123], and sobering-up shelters [124], as well as community-controlled residential alcohol treatment services [125, 126]. It could be argued that Woolworths ignored or, at worst, silenced the Aboriginal voice in this debate. This is significant because the Uluru Statement from the Heart along with the upcoming First Nations’ Voice Referendum exemplify the contemporary expectations for commercial entities to empower, recognise and promote self-determination for Aboriginal and Torres Strait Islander peoples [127].

### Implications for public health and advocacy

A key finding of this case study is that a well organised coalition of Aboriginal and non-Aboriginal health advocates implementing a sustained, multipronged campaign of media, legal and corporate strategies can successfully overcome powerful commercial interests, particularly when it comes to protecting Aboriginal health and wellbeing. A unique strategy used by the advocates was shareholder action by informing Woolworths’ investors of the health and wellbeing implications of the proposed store and corresponding with the Woolworths board to keep them accountable for the company’s decisions. The corporate campaigning and the media strategy, according to the participants, was instrumental in the success of the campaign. This is supported by research highlighting that many employees of investment institutions believe advocacy is fundamental in driving decision-making and that media coverage of issues is “an important lever for change” (p.9, [128]). The role of investment institutions are important in influencing businesses and business practices, particularly in recent decades [52]. Similarly, when it comes to mobilising change within government regarding Aboriginal health, civil society can be a powerful collective voice for building a groundswell for change [129], as demonstrated in the present case study. By providing voice and agency to members of local Aboriginal communities impacted by alcohol, along with a nation change.org petition and open letter published in a major Australian newspaper, public awareness grew dramatically, followed by a groundswell of national support. Furthermore, the fact that the resistance to Woolworths’ proposal was being led by Aboriginal health organisations reinforces previous findings surrounding Aboriginal services operating in direct resistance to commercial interests and promoting self-determination [130–132].

The political dynamics observed in this case study underscore the need to reform the management of industry-government relationships. The political pressure and lobbying by the alcohol industry, both within the NT and at the Australian federal level, has been well documented [22, 25, 26, 106, 133]. The alcohol industry has provided political donations to the major parties in the NT, the Country Liberal Party and the Australian Labor Party in the past [134]. A 2018 inquiry into options for reform of political funding and donations in the NT found that the alcohol industry has had strong influence over government decision-making over the last 10 years [135]. Numerous submissions to this inquiry called for banning donations from harmful industries, including alcohol [135]. However, such reforms have not been introduced at either federal or state/territory levels in Australia [136].

The rapid changes in alcohol policies during this case study highlight the politicization of alcohol consumption. The primary policies in the NT surrounding alcohol focus on regulating its availability [19]. The Alcohol Harm Minimisation Action Plan 2018 made 220 recommendations for alcohol policy reform, of which 219 were passed, including the introduction of minimum unit pricing [137] and the re-introduction of the banned drink register [138], in order to reduce alcohol consumption [139]. However, it has been argued that many of the strategies in place have an implicit focus on alcohol consumption among Aboriginal people, without addressing the fundamental health context of colonisation including dispossession, oppression, disempowerment, and trauma [19, 140, 141]. Aboriginal community-controlled organizations have embedded systems in place that represent their local communities therefore such organisations provide meaningful insight in developing health equity focused alcohol-policies [122, 142, 143]. It is important that a diverse range of Aboriginal groups be included in the development of policies to reduce alcohol-related harm.

Participants in this case study stressed that the proposed alcohol store would not only impact the health of Aboriginal residents but the health of Territorians more broadly. The health risks were underplayed in the public discourse by Woolworths and the NT government throughout legal proceedings, reinforcing the normalization of alcohol in Darwin. The purpose of using such strategies for commercial gain is to deflect attention away from the industry’s involvement in causing harm [112]. However, evidence clearly demonstrates that an increase in the density of alcohol supplied is associated with increases in alcohol-related violence [96], including domestic and family violence [94, 95]. There is strong evidence that high levels of alcohol consumption in the NT are driving numerous health and social problems [18, 144, 145] and that Territorians are the highest consumers of alcohol than any other state or territory in Australia [68, 99]. The current situation in Alice Springs (in central NT), following the lifting of alcohol-bans in July 2022 for the first time in 15 years [146], saw a 38% increases in assaults and 62% increases in domestic abuse [147], highlighting how alcohol supply directly increases alcohol-related harm.

Several lessons can be drawn from this case study for other social justice and health equity advocacy campaigns. The coalition of actors opposing the Dan Murphy’s store were unified in their message and used numerous strategies to raise awareness and garner public support against Woolworths’ proposal. The literature on successful advocacy coalitions highlights the importance of having a cohesive, collective voice; policy ‘champions’, civil society mobilisation and institutional leadership [129, 148, 149], all of which were present in this case. The campaign against the Dan Murphy’s store also reinforces the effectiveness of several well documented advocacy strategies, including strategic framing, targeted advertising in prominent outlets, shareholder and investor lobbying, legal action and presenting the evidence [150–153]. This case study further emphasised the effectiveness of corporate campaigning, employing shareholder action as a powerful strategy in public health advocacy.

### Strengths and limitations

A strength of this case study was the involvement of Aboriginal people and organisations. The research was undertaken with the support of the Aboriginal Medical Services Alliance NT, the peak Aboriginal health body in the NT, and the research team included two Aboriginal researchers from Darwin (BC and YP). Another strength was the application of an adapted corporate health impact assessment framework [59], that has been demonstrated as a useful tool in previous case studies assessing the impact of corporations, including alcohol companies [52, 59, 61, 62]. The framework enabled an understanding of the social-economic and political conditions within the NT that set the context for the case study. CDoH scholars argue that it is important to document both commercial activities and the socio-economic-political conditions in which they operate as business operations are a symptom of the regulatory environment [154]. A limitation of this case study was that no industry representatives were interviewed. However, prior research reinforces that the assessment of a large corporation and its health impacts does not depend on industry engagement [155]. Further, industry perspectives were included through the collection of media articles and documents from Woolworths’ website.

## Conclusion

This case study has documented the actions of Woolworths in their pursuit to build the largest alcohol outlet in the NT and reflects on the strategies used by civil society advocates in resistance to Woolworths commercial interests. Woolworths did not undertake adequate consultation with Aboriginal and non-Aboriginal health organisations and, while not admitting to the potential for harm, ultimately abandoned their proposal. Multiple advocacy strategies were deployed by a coalition of Aboriginal and non-Aboriginal organisations and Aboriginal Elders to resist the construction of the alcohol megastore. Similar coalitions and strategies may be useful in future advocacy campaigns to safeguard Aboriginal health and wellbeing from harmful commercial interests.

## Data Availability

The qualitative interview data and Freedom of Information documents in this study is not publicly available. All other data is publicly available.

## Abbreviations

AMSANT: Aboriginal Medical Services Alliance Northern Territory
AGM: Annual General Meeting
CDoH: Commercial Determinants of Health
CDoIH: Commercial Determinants of Indigenous Health
LMIC: Lower-and-middle-income country
FARE: Foundation for Alcohol Research and Education
HSIA: Health and Social Impact Assessment
NT: Northern Territory
NTCAT: Northern Territory Civil and Administrative Tribunal
RAP: Reconciliation Action Plan

## Footnotes

1 The Indigenous population in the NT is mostly Aboriginal peoples, therefore hereafter we will respectfully use the term Aboriginal when referring to Indigenous peoples in the NT.
